# Therapy‐Related Acute Myeloid Leukemia Following Plasma Cell Leukemia: A Case Report and Literature Review

**DOI:** 10.1002/ccr3.71896

**Published:** 2026-02-02

**Authors:** Songdi Chen, Junqing Hu, Qian Zhang, Luling Mao, Qian Jiang, Chuyun Qian, Weize Zhang, Keting Jin, Jianhu Li, Ji‐nuo Wang, Yi Zhao

**Affiliations:** ^1^ Department of Hematology The First People's Hospital of Yuhang District (Zhejiang University School of Medicine First Affiliated Hospital Liangzhu Hospital) Hangzhou China; ^2^ Clinical Laboratory of the First People's Hospital of Yuhang District Hangzhou China; ^3^ Department of Hematology, the First Affiliated Hospital Zhejiang University School of Medicine Hangzhou China; ^4^ Bone Marrow Transplantation Center of the First Affiliated Hospital Zhejiang University School of Medicine Hangzhou China; ^5^ Institute of Hematology Zhejiang University Hangzhou China

**Keywords:** azacitidine, multiple myeloma, plasma cell leukemia, therapy‐related acute myeloid leukemia, venetoclax

## Abstract

With the improvement of survival in multiple myeloma (MM), therapy‐related acute myeloid leukemia (t‐AML) has emerged as a clinically relevant second primary malignancy (SPM). We report a case of MM evolving into t‐AML after multi‐agent chemotherapy and review the literature on therapy‐related leukemias in MM. We report a case of a patient diagnosed with primary plasma cell leukemia (IgG‐λ type, R‐ISS stage III) who achieved complete remission following maintenance therapy with daratumumab, lenalidomide, and dexamethasone after receiving a treatment regimen based on proteasome inhibitors. The patient progressed to therapy‐related acute myeloid leukemia 18 months later, and we present the clinical features. Additionally, we conducted a literature review. Given the patient's age and debilitated physical condition, treatment with azacitidine combined with venetoclax was administered. Following the treatment, the patient developed grade IV post‐chemotherapy myelosuppression complicated by infection and extensive ischemic stroke. Despite aggressive supportive care, the patient's condition continued to deteriorate and he succumbed in August 2025. This case illustrates the leukemogenic risk of cytotoxic exposure in MM, highlights the adverse genetic profile of therapy‐related AML, and emphasizes the need for vigilant monitoring and preventive strategies in long‐term MM survivors.

## Key Clinical Message

The case highlights how high‐risk pPCL patients progress to t‐AML following multi‐agent cytotoxic therapy. Furthermore, this case emphasizes the complex interplay between cytotoxic therapy and high‐risk disease biological characteristics in the induction of t‐AML and underscores the necessity of careful monitoring and risk mitigation for long‐term MM survivors.

## 1 Introduction

Therapy‐related myeloid neoplasms (t‐MNs) are among the most devastating late complications of successful cancer therapy, representing approximately 10% to 20% of newly diagnosed AML/MDS [1]. With the advent of proteasome inhibitors, immunomodulatory drugs (IMiDs), and monoclonal antibodies, patients with multiple myeloma (MM) are surviving longer, yet face an increasing risk of second primary malignancies (SPMs), including t‐AML and, less frequently, therapy‐related acute lymphoblastic leukemia (t‐ALL) [2, 3]. Despite these advancements, therapy‐related leukemias remain aggressive, with poor overall survival often measured in months [2]. We report here a case of primary plasma cell leukemia (pPCL) evolving into t‐AML during lenalidomide maintenance therapy without prior transplantation, highlighting underlying etiological factors and the therapeutic challenges in such high‐risk settings.

## 2 Case History and Examination

**FIGURE 1:**
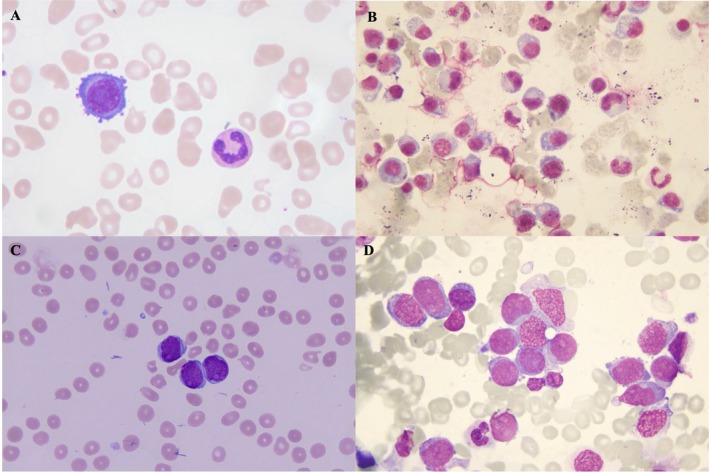
Bone marrow and peripheral blood smears of the patient at different disease stages. (A) Peripheral blood smear showing abnormal circulating plasma cells (Wright–Giemsa stain, ×1000). (B) Bone marrow smear demonstrating atypical plasma cell infiltration (Wright–Giemsa stain, ×1000). (C) Peripheral blood smear with numerous circulating myeloblasts at AML diagnosis (Wright–Giemsa stain, ×1000). (D) Bone marrow smear revealing extensive proliferation of myeloblasts (Wright–Giemsa stain, ×1000).

A 70‐year‐old man was admitted in December 2022 with newly detected hypergammaglobulinemia during routine examination. Past medical history included hypertension, type 2 diabetes mellitus, and chronic hepatitis B. Physical examination revealed pallor without lymphadenopathy, hepatosplenomegaly, or peripheral edema. Laboratory evaluation showed hemoglobin 105 g/L and serum globulin 101.8 g/L with albumin 27.3 g/L, while renal function, calcium, and LDH were normal. Peripheral blood smear showed 14% abnormal cells (Figure 1A). Serum immunofixation electrophoresis demonstrated IgG‐λ monoclonal protein (M‐protein 73.4 g/L). Serum free light chain assay showed κ 23.4 mg/dL, λ 1930 mg/dL, ratio 0.01. β2 microglobulin was 4.11 mg/L. Bone marrow examination revealed 42% plasma cells (Figure 1B) with abnormal immunophenotype (CD38^+^, CD138^+^, CD56^+^, CD200^+^, cytoplasmic λ). No morphologic dysplasia, increased blasts, or abnormal maturation were observed. FISH analysis demonstrated high‐risk abnormalities: t(4;14), 1q21 gain, del(13q), and del(17p). PET‐CT revealed multiple lytic lesions of spine bones. The diagnosis of primary PCL was established.

**FIGURE 2:**
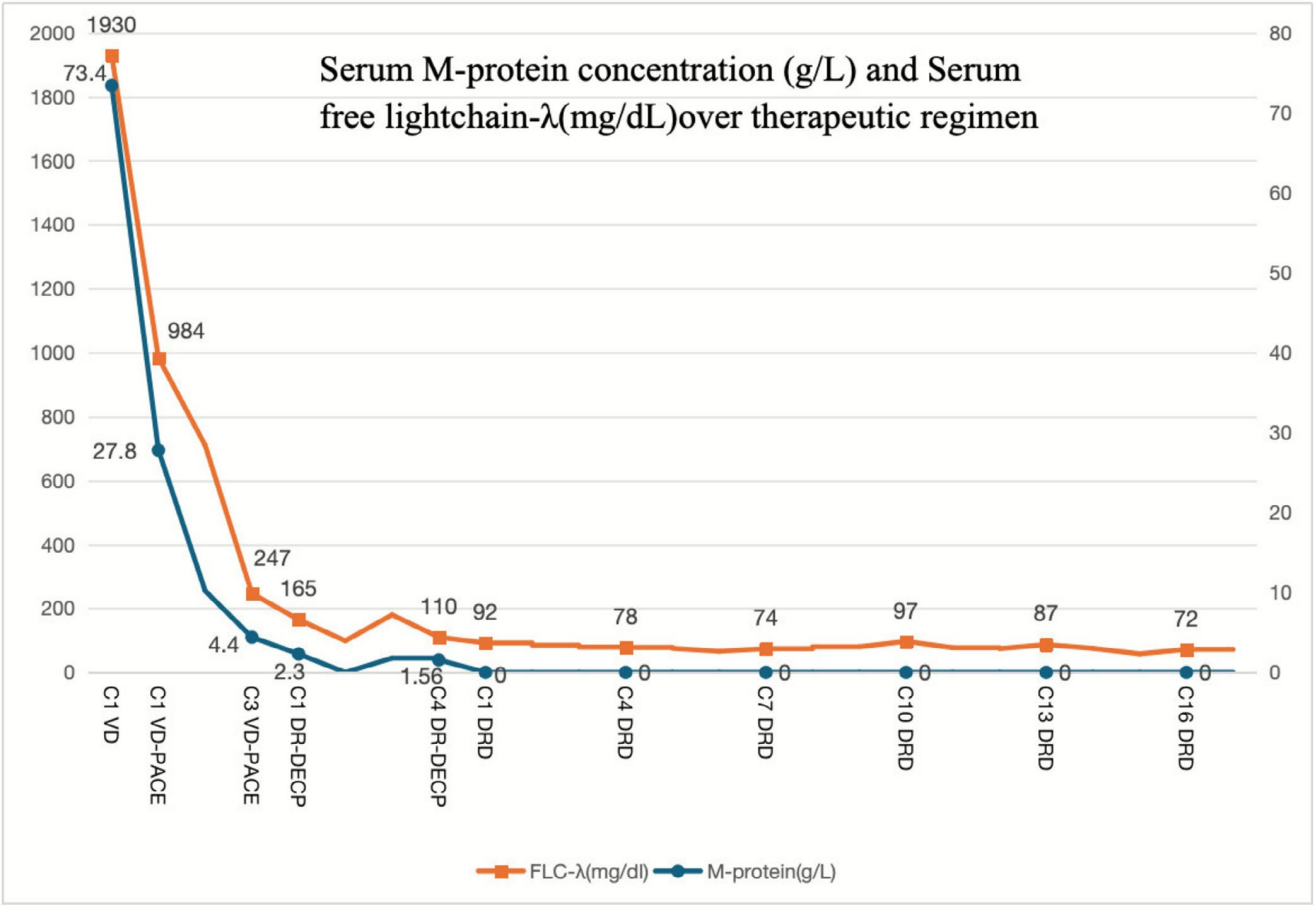
The longitudinal changes in M‐protein (g/L) and free light chain‐λ (mg/dL) levels in relation to the treatment.

The patient initially received VD (bortezomib 2.45 mg d1, d4, d8, d11; dexamethasone 20 mg d1‐2, d4‐5, d8‐9, d11‐12), followed by three cycles of dose‐reduced VD‐PACE (bortezomib 2.55 mg d1, d8, d15, d22; dexamethasone 20 mg d1‐4; cisplatin 10 mg d1‐4; liposomal doxorubicin 20 mg d5; cyclophosphamide 0.4 g d1‐4; etoposide 38 mg d‐4). Due to severe myelosuppression and pulmonary infection, he transitioned to 4 cycles of dose‐reduced regimen (daratumumab 1184 mg qw for 8 weeks, q2w for 16 weeks, lenalidomide 10 mg qn d1‐21, dexamethasone 20 mg d2‐5, cisplatin 10 mg d2‐5, cyclophosphamide 0.4 g d2‐5, and etoposide 38 mg d2‐5). After eight cycles, complete remission (CR) was achieved. From October 2023 to May 2025, he received DRD (Daratumumab 1184 mg q4w, lenalidomide 10 mg qod d1‐21, and dexamethasone 20 mg d1) maintenance for 18 months, during which he remained in sustained CR (Figure 2) and MRD negativity. Due to significant myelosuppression and poor tolerance, lenalidomide was administered at a reduced dose of 10 mg every other day as maintenance therapy, and 100 mg aspirin was given per day to inhibit platelet aggregation.

## 3 Investigations and Treatment

In June 2025, he developed fatigue and dizziness. Blood counts showed leukocytosis (WBC 30.74 × 10^9^/L), anemia (Hb 53 g/L), and thrombocytopenia (PLT 37 × 10^9^/L) with 65% circulating blasts (Figure 1C). Bone marrow revealed 78% myeloblasts consistent with AML (Figure 1D). Flow cytometry confirmed blasts expressing CD117, CD34, CD33, CD13, HLA‐DR, CD123, and CD56. Cytogentics showed 44,X,Y,‐7 [9]/46,Y,add(X)(p22.2),?inv.(1)(p11q25)[1]. Next‐generation sequencing revealed mutations in ASXL1 (VAFs 43.76%), KRAS (VAFs 29.37%), PTPN11 (VAFs 12.97%), and an ETV6‐DUSP16 fusion. The patient was diagnosed as a myeloid neoplasm post cytotoxic therapy (pCT) according to the 5th edition of the WHO classification in 2024 and classified as intermediate‐risk based on the 2024 ELN criteria [4].

Given his age and frailty, the patient received azacitidine (130 mg d1–7) plus venetoclax (100 mg d1, 200 mg d2, 400 mg d3–15). He developed persistent severe neutropenia, thrombocytopenia, and recurrent febrile episodes, treated with meropenem, caspofungin, and supportive transfusions.

## 4 Outcome and Follow‐Up

**FIGURE 3:**
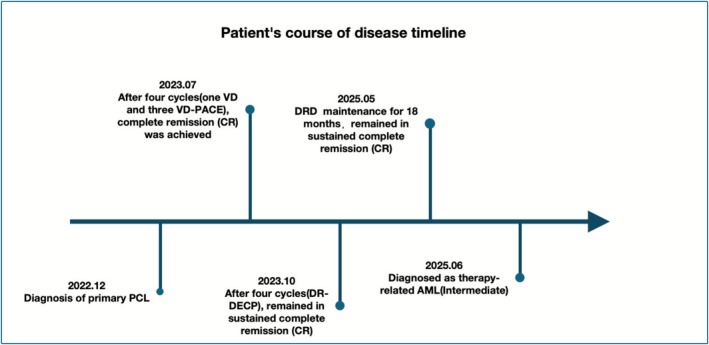
Visual timeline summarizing treatment, responses, and onset of t‐AML.

On July 31st, 2025, he experienced sudden loss of consciousness and left‐sided hemiplegia. Computerized tomography (CT) revealed a massive right hemispheric ischemic stroke. Despite supportive care, his condition deteriorated, and he died in August 2025. Figure 3 shows the timeline of his whole disease progress, including treatment, responses, and onset of t‐AML.

## 5 Discussion

Multiple myeloma (MM) is a malignant clonal proliferation of plasma cells. Over the past two decades, the incorporation of proteasome inhibitors, immunomodulatory drugs, anti‐CD38 monoclonal antibodies, and risk‐adapted autologous stem‐cell transplantation (ASCT) has substantially prolonged survival, but has also made second primary malignancies (SPMs)—particularly therapy‐related myeloid neoplasms (t‐MN)—a more visible late complication in the MM population [5]. Across contemporary cohorts, alkylating‐agent exposure remains the most consistent risk factor for t‐MN after MM therapy: historical oral melphalan regimens were strongly implicated in leukemic transformation, and high‐dose melphalan used for ASCT conditioning continues to contribute to risk in modern series [2, 5]. Our patient did not undergo ASCT because of age and preference, which removes a major alkylator‐intensive exposure from consideration.

The relationship between lenalidomide maintenance and hematologic SPMs (including AML/MDS as well as ALL) has been observed in trials and registries, especially in patients with prior melphalan exposure [3, 6]. At a mechanistic level, lenalidomide's cereblon‐dependent degradation of IKZF1/3 may inadvertently select for TP53‐mutated hematopoietic stem/progenitor cells, thereby fostering clonal evolution under long‐term exposure [3, 7]. In our case, lenalidomide was ultimately reduced to 10 mg every other day (qod) for maintenance because of significant myelosuppression and intolerance, and the cumulative duration at standard doses was limited. These features, coupled with the absence of ASCT, further weaken a direct causal attribution of t‐AML to lenalidomide alone in this individual patient. By contrast, the patient did receive multi‐agent cytotoxic therapy (including a VDPACE‐based regimen) early in the disease course, which—together with patient‐intrinsic factors—may have contributed to leukemogenesis.

With respect to anti‐CD38 therapy, daratumumab‐containing combinations improve depth and durability of response in both newly diagnosed and relapsed settings (e.g., MAIA, ALCYONE, POLLUX) and are integral to contemporary MM backbones [8, 9, 10]. A recent systematic review/meta‐analysis has not demonstrated an increased incidence of hematologic SPMs attributable to anti‐CD38 antibodies; nonetheless, continued long‐term follow‐up is warranted [11]. In our case, the use of daratumumab likely facilitated attainment and maintenance of deep remission without clearly increasing leukemogenic risk based on current evidence.

An additional layer of complexity in this case is the patient's initial diagnosis of primary plasma cell leukemia (pPCL)—an ultra–high‐risk plasma cell neoplasm defined by circulating plasma cells and characterized by enrichment for adverse cytogenetics such as t(4;14), del(17p), and 1q gains/amplifications, as well as a propensity for early relapse and short survival despite novel agents [12, 13]. pPCL often exhibits distinct immunophenotypic features, notably reduced or absent CD56 (NCAM) expression and, in some series, increased CD44, reflecting altered adhesion and impaired marrow anchoring; these changes are associated with enhanced circulation of tumor cells and frequent extramedullary spread [12, 14, 15]. Standard myeloma regimens are frequently insufficient for durable control; when feasible, intensive multi‐agent induction followed by consolidative transplantation or enrollment in clinical trials is advocated to maximize the chance of remission [12, 13]. In our patient, intensive induction (including VDPACE) and subsequent anti‐CD38/IMiD‐based therapy achieved a complete remission of the plasma cell clone; however, the biologic aggressiveness of pPCL necessitated substantial cytotoxic exposure up front, complicating causal inference when t‐AML later emerged.

Whether pPCL predisposes to therapy‐related leukemogenesis is not established. Large cohort analyses of t‐MN after MM generally do not stratify by pPCL versus typical MM, and available data suggest that cytotoxic exposures and baseline adverse genetics are the principal drivers of t‐MN risk [2]. Thus, in our case, it is most plausible that the combination of high‐risk baseline biology (including t(4;14)/1q/17p abnormalities typical of pPCL) and multi‐agent cytotoxic exposure, even though lenalidomide maintenance is at a low alternate‐day dose, contributed to leukemogenesis.

Recent cohort studies have reported that mutations in genes such as ASXL1, KRAS, PTPN11, as well as chromosomal abnormalities (e.g., del(7), preciously denoted as “‐7”), drive clonal hematopoiesis of indeterminate potential (CHIP) [16]. Clonal hematopoiesis (CH) with a variant allele frequency (VAF) > 2% is positively correlated with the risk of t‐MN, and this risk increases with the total number of mutations and the size of the clone population [17]. Furthermore, the median interval of 3–5 years between MM diagnosis and the development of t‐MN, with shorter latencies observed in patients exposed to intensive cytotoxic regimens or harboring high‐risk MM cytogenetics [18, 19, 20]. In this context, our patient's 3‐year interval from MM to t‐AML aligns with the accelerated subset described in modern MM‐specific cohorts. The combination of pPCL‐associated high‐risk features and multi‐agent chemotherapy likely contributed to this shortened leukemogenic trajectory.

Emerging genomic studies have linked monocytic differentiation and activation of the RAS/MAPK pathway with reduced sensitivity to venetoclax; RAS‐pathway mutations such as KRAS may therefore contribute to venetoclax resistance either directly through anti‐apoptotic reprogramming or indirectly by promoting monocytic lineage features [21, 22]. In our case, the immunophenotype and cytochemical data support a non‐monocytic AML with maturation phenotype, but the presence of a KRAS mutation could still have impaired venetoclax efficacy via RAS‐driven survival signaling. In parallel, exposure to topoisomerase II inhibitors, such as etoposide used in our patient's induction regimens, has been associated with early‐onset therapy‐related myeloid neoplasms, typically arising within 1–3 years and often accompanied by cooperating RAS‐pathway lesions. The coexistence of these mutations together with prior topo‐II inhibitor exposure may therefore have synergistically accelerated leukemogenesis and may contribute to the poor response to azacitidine–venetoclax in this patient.

Therapeutically, options for older or frail patients with t‐AML remain limited. Azacitidine plus venetoclax improved response and survival versus azacitidine alone in VIALE‐A and is the current standard for many unfit AML patients [23]. Nevertheless, outcomes in t‐AML are consistently inferior to de novo AML—particularly with adverse cytogenetics or lesions such as ASXL1 and TP53—and therapy is frequently complicated by prolonged cytopenias and serious infections, as illustrated by this case [3, 24]. These challenges underscore the need for risk‐mitigation strategies (e.g., minimizing alkylator exposure when feasible, judicious IMiD use and dosing, MRD‐guided de‐escalation studies) and novel approaches that preserve MM control without materially increasing leukemogenic risk.

In summary, this case highlights the complex interplay between intensive therapy and aggressive baseline disease biology in the development of therapy‐related AML. It emphasizes the importance of balancing effective myeloma control with long‐term leukemogenic risks and the need for vigilant monitoring in high‐risk patients such as those with primary plasma cell leukemia.

## 6 Conclusion

We describe a rare case of therapy‐related AML following treatment of primary plasma cell leukemia. Despite reduced dose lenalidomide maintenance and absence of transplantation, the patient developed AML with monosomy 7 and adverse mutations and died rapidly despite azacitidine‐venetoclax. This case underscores the leukemogenic role of multi‐agent cytotoxic exposure on a background of aggressive plasma cell biology, the uncertain independent role of pPCL, and the urgent need for preventive strategies and novel therapeutic approaches.

## Author Contributions

**Songdi Chen:** data curation, investigation, visualization, writing – original draft. **Qian Zhang:** formal analysis, investigation. **Junqing Hu:** data curation, investigation, visualization. **Luling Mao:** data curation, investigation. **Qian Jiang:** data curation, investigation. **Chuyun Qian:** data curation, investigation. **Weize Zhang:** data curation, investigation. **Keting Jin:** data curation, investigation. **Jianhu Li:** data curation, investigation, visualization. **Ji‐nuo Wang:** conceptualization, funding acquisition, supervision, visualization, writing – original draft, writing – review and editing. **Yi Zhao:** conceptualization, funding acquisition, project administration, supervision, writing – review and editing.

## Funding

This work was supported by the National Natural Science Foundation of China (Grant No. 82000221 to J.W.) and the Natural Science Foundation of Zhejiang Province (Grant No. LY22H080004 to Y.Z.).

## Consent

This study was conducted in accordance with the principles of the Declaration of Helsinki. Written informed consent was obtained from all patients for publication.

## Conflicts of Interest

The authors declare no conflicts of interest.

## Data Availability

The data that support the findings of this study are available from the corresponding author upon reasonable request.
